# Supplementary material for: Relationship between cricket participation, health and well-being: scoping review protocol
Source: BMJ Open. 2019 Nov 10;9(11):e032070. doi: 10.1136/bmjopen-2019-032070 (PMC6858230; doi:10.1136/bmjopen-2019-032070)
Supplement: Supplementary data [file bmjopen-2019-032070supp001.pdf]

Appendix ##: Example of full search strategy.

Medline and PubMed:
*cricket** NOT (*cadaver* or "in situ" or "in vitro" or animals or insects*)
