# Supplementary material for: Relationship between cricket participation, health and well-being: scoping review protocol
Source: BMJ Open. 2019 Nov 10;9(11):e032070. doi: 10.1136/bmjopen-2019-032070 (PMC6858230; doi:10.1136/bmjopen-2019-032070)
Supplement: Author's manuscript [file bmjopen-2019-032070.draft_revisions.pdf]

BMJ Open is committed to open peer review. As part of this commitment we make the peer review history of every article we publish publicly available.

When an article is published we post the peer reviewers' comments and the authors' responses online. We also post the versions of the paper that were used during peer review. These are the versions that the peer review comments apply to.

The versions of the paper that follow are the versions that were submitted during the peer review process. They are not the versions of record or the final published versions. They should not be cited or distributed as the published version of this manuscript.

BMJ Open is an open access journal and the full, final, typeset and author-corrected version of record of the manuscript is available on our site with no access controls, subscription charges or pay-per-view fees (http://bmjopen.bmj.com).

If you have any questions on BMJ Open's open peer review process please email info.bmjopen@bmj.com

BMJ Open

The relationship between cricket participation, health, and wellbeing: A scoping review protocol

Journal:	*BMJ Open*
Manuscript ID	bmjopen-2019-032070
Article Type:	Protocol
Date Submitted by the Author:	03-Jun-2019
Complete List of Authors:	Bullock, Garrett; University of Oxford, Nuffield Department of Orthopaedics, Rheumatology, and Musculoskeletal Sciences<br>Panagodage-Perera, Nirmala ; University of Oxford, Arthritis Research UK Centre for Sport, Exercise & Osteoarthritis, Nuffield Department of Orthopaedics, Rheumatology & Musculoskeletal Sciences<br>Murray, Andrew; University of Edinburgh, Physical Activity for Health Research Centre<br>Arden, Nigel; Nuffield Department of Orthopaedics, ;<br>Filbay, Stephanie; University of Oxford, Arthritis Research UK Centre for Sport, Exercise & Osteoarthritis, Nuffield Department of Orthopaedics, Rheumatology & Musculoskeletal Sciences
Keywords:	Physical Activity, Health Related Quality of Life, Injury, MENTAL HEALTH

SCHOLARONE™
Manuscripts

The relationship between cricket participation, health, and wellbeing: A scoping review protocol

Garrett S. Bullock PT, DPT,[1,2] Nirmala K. Panagodage Perera, MMed, PhD,[1,2,3,4] Andrew Murray, MD,[5] Nigel K. Arden, MD, FRCP,[1,2] Stephanie R. Filbay, B.Phty (Hons), PhD[1,2]

1. Centre for Sport, Exercise and Osteoarthritis Research Versus Arthritis; Oxford, United Kingdom
2. Nuffield Department of Orthopaedics, Rheumatology and Musculoskeletal Sciences, University of Oxford, Oxford, United Kingdom
3. Division of Physiotherapy, Department of Medical and Health Sciences, Linköping University, Sweden.
4. Latrobe Sports and Exercise Medicine Research Centre, College of Science, Health and Engineering, Latrobe University, Bundoora VIC 3086, Australia
5. Sport and Exercise, University of Edinburgh, Edinburgh, UK

Corresponding Author

Garrett S. Bullock PT, DPT
Nuffield Department of Orthopaedics, Rheumatology, and Musculoskeletal Sciences
University of Oxford
B4495
Oxford, United Kingdom
OX3 7LD
Telephone: (865) 227-374
Fax: (865) 768-876
garrett.bullock@ndorms.ox.ac.uk

Key Words: Physical Activity, Health Related Quality of Life, Injury, Mental Health, Musculoskeletal

Word Count: 1620

Competing Interests

All authors affirm that they have no involvement with any commercial organization that has a direct financial interest in any matter included in this manuscript.

For peer review only - http://bmjopen.bmj.com/site/about/guidelines.xhtml

For peer review only

Abstract

Introduction: Cricket is a popular sport played by 2.5 billion people of all ages and abilities. Participation in cricket has potential to both positively and negatively impact health and wellbeing. However, due to no literature review in this area, the relationship between cricket participation, health and wellbeing remains unclear. Therefore, the aim of this scoping review is to i) investigate the relationship between cricket participation, health and wellbeing and ii) identify the research gaps related to cricket, health and wellbeing

Methods and analysis: Methodology of this paper was informed by previous scoping review protocols and best practice methodological frameworks. Medline, CINAHL, Embase, Scopus, PsycINFO, SPORTDiscus, Cochrane Library, EBSCO, Web of Science and PEDro, and grey literature sources (Google Scholar, Clinicaltrials.gov, ISRCTN Registry and Proquest) will be systematically searched. Studies that assess a construct related to health and/or wellbeing in current and/or former cricketers will be eligible. Two reviewers will independently screen full-texts of identified studies for eligibility, and perform data extraction. Results will be presented in tabular and graphical form and reported descriptively.

Ethics and dissemination: This research is exempt from ethics approval due to the data is available through published and public available resources. Results will be published in a peer-reviewed sports and exercise medicine journal regardless of positive or negative findings. Through a multi-modal approach, stakeholder groups including cricketers, practitioners and policy makers will be informed. There findings will inform clinical decision making, policy changes, and future research agendas.

Key Words: Physical Activity, Health Related Quality of Life, Injury, Mental Health, Musculoskeletal

Strengths and Limitations

For peer review only - http://bmjopen.bmj.com/site/about/guidelines.xhtml

- Scoping reviews are a scientifically validated method to answer broad research questions, and is the best methodology to provide an overview of all literature investigating the relationship between cricket participation, health and wellbeing.
- This scoping review will include grey literature to increase the scope and breadth of the review and screening process.
- Individual article data will be meta-aggregated to explore emergent themes, with potential to provide new insights and inform future research
- Specific articles cannot be analyzed for methodological risk of bias, decreasing the interpretability of the results.

For peer review only

For peer review only - http://bmjopen.bmj.com/site/about/guidelines.xhtml

Introduction

Cricket is a popular sport with approximately 2.5 billion people of all ages and abilities participating.[1] Cricket is played by 1.4 million people in Australia,[2] nearly 300,000 people in the United Kingdom,[3] and over 5 million people in India.[4] Further, cricket is popular among youth,[5 6] many of whom continue to play cricket into adulthood.[7] Cricket has also become increasingly popular among females with more than 27% of all Australian cricketers being female.[2] Cricket is played with 11 individuals per team, over 5 days (test cricket), one day (50 overs) or over four hours (Twenty20). The 2011 Compendium of Physical Activities list cricket as a sport that can provide moderate-intensity physical activity.[8] Regular physical activity is an important determinant of general health, life expectancy[9] and overall wellbeing.[10]

Over 31% of all adults worldwide are physically inactive, with physical inactivity levels ranging from 17% in Asia, to 43% in North America.[11] To counteract inactivity, sports participation is promoted.[12] Sport participation provides opportunities to be physically active across the lifespan.[13 14] Cricket participation can improve fitness[15 16] and strength,[16] and has psychological benefits for participants[17-19] including improved self-esteem, social connections and overall wellbeing.[17] Mental health and health-related quality of life (HRQoL) is higher in cricketers compared to the general population.[20 21] However, cricket participation is associated with injury[22-25] which can result in persistent joint pain and post-traumatic osteoarthritis.[26] Specifically, injury incidences has been reported to be up to 53 injuries per 10,000 athlete exposures,[27] with former cricketer reporting greater osteoarthritis compared to former rugby players.[28] Further, some cricketers experience increased levels of stress[29 30] and depression,[31 32] which can negatively impact HRQoL,[26 33 34] Thus, cricket participation may have both positive and negative impacts on health and wellbeing. Due to

For peer review only - http://bmjopen.bmj.com/site/about/guidelines.xhtml

the high rate of global physical inactivity,[11] the worldwide popularity of cricket,[2-4] and its viability as an outlet for physical activity across the lifespan,[13 14] information regarding the potential risks and benefits of participation in specific sports is needed to enable informed decision making for participants.

The link between sport participation and beneficial health outcomes has been synthesised in a previous systematic review[35]; however, the relationship between cricket, health and wellbeing has not been investigated. Thus, there is a need to map the current evidence related to cricket, health and wellbeing, and identify key research priorities. This overview would also enable key stakeholders (including cricket participants, health professionals and sporting bodies) to make evidence informed decisions relating to cricket participation. The aim of this scoping review is to (1) investigate the relationship between cricket participation, health and wellbeing at all ages and standards-of-play; (2) identify research gaps in the existing literature on cricket, health and wellbeing.

Methods

The purpose of a scoping review is to describe all available evidence underpinning a given research question drawing upon research from all possible sources, consequently scoping reviews are broad in nature. The framework adopted for this scoping review follows existing best practice methodology.[36-40] The methodology was guided by the recommended five-stage process: identify the research question; identify relevant studies; select articles using *a priori* inclusion/exclusion criteria; chart data; collate, summarize, and report results.[36]

Stage 1: Identify the research question

For peer review only

The general research question was developed through exploration of the literature, multi-disciplinary group discussions and collaborating with experts in cricket. To reflect the context, content and the population included in the review,[37] the following broad research question was proposed: *what is known about the relationship between cricket participation, health and wellbeing?*

Stage 2: Identify relevant studies

A preliminary search to identify key words and index terms

A preliminary search was conducted on the major clinical and grey literature databases.[36] Databases included Medline, Google Scholar, and ProQuest Dissertations and Theses Global. Consistent with previous studies[36] exploratory search terms were kept broad, to be as inclusive as possible. Search terms included "*cricket*," "*health*," and "*review*." The exploratory search found 37 articles in Medline. The first 200 articles in Google Scholar were searched. Twenty-eight articles were identified as pertinent from Medline and Google Scholar. No relevant articles were found in ProQuest. These 28 articles references were then searched for further relevant articles.

The titles and abstracts of these 28 articles were then analysed for relevant search terms. The preliminary search identified a large number of irrelevant studies involving cricket insects, cadaveric, or in vitro investigations, consequently the search terms were updated to exclude articles with cadaver* or "in situ" or "in vitro" or insects in the title and/or abstract.

Search strategy

Ten databases (Medline, CINAHL, Embase, Scopus, PsycINFO, SPORTDiscus, Cochrane Library, EBSCO, Web of Science and PEDro) will be electronically searched. Google

For peer review only - http://bmjopen.bmj.com/site/about/guidelines.xhtml

Scholar, Clinicaltrials.gov, ISRCTN Registry and Proquest Dissertation and Theses will be searched for grey literature. The search strategy will be as follows, *"cricket** NOT (*cadaver* or "in situ" or "in vitro" or animals or insects*)". ***Articles** will be tracked in* EndNote® X9 (Clarivate Analytics, 2018).

Study eligibility criteria

To be eligible for inclusion in this review, articles must meet the following criteria:

i) Assesses a construct related to health (e.g. injury, pain, physiological function, physical activity, tobacco use, alcohol use, body mass index, nutrition, diabetes, cardiovascular disease) and/or wellbeing (e.g. mental health, depression, mood, anxiety, HRQoL, resilience) in current and/or former cricketers (of any age, sex or competition level);

ii) Primary research studies, reviews, meta-analyses, guidelines or grey literature (including unpublished and ongoing trials, annual reports, dissertations and conference abstracts);

iii) Human studies;

iv) Due to the language proficiency of the authors, only articles published in English will be eligible.

The following constructs were considered to be of little relevance to health or wellbeing, therefore, articles reporting only these construct will be in-eligible for review:

- Cricket performance parameters (e.g. bowling speed, wins and losses, and bowling average);
- Biomechanics (force, torque, kinematics and electromyography);
- Joint range of motion/flexibility.

For peer review only - http://bmjopen.bmj.com/site/about/guidelines.xhtml

Additional exclusion criteria:

- Editorials, periodicals, letters to the editor;
- Cadaveric, or in situ models studies

Stage 3: Study selection

Titles and abstracts will be screened by the lead author (GSB) for eligibility, and full-text articles will be retrieved and screened by the same author (GSB) against the inclusion/exclusion criteria. A second author (NKPP) will complete the same screening process on a random sample of 10% of the articles.[36] Any title and abstract screening disputes will be resolved through the consensus of the two authors. If concordance is less than 90%, the full title and abstract screening will be performed by the second author (NKPP). Following title and abstract screening, the full text of all potentially eligible articles will be retrieved. If a full-text article cannot be retrieved following consultation with a librarian, it will be excluded from the review.[36] If there are any discrepancies following full-text screening, a third author (SRF) will arbitrate all disputes, and decide on final article inclusion.

Stage 4: Data extraction

Data extraction procedures with follow best systematic review practice guidelines.[41] Data will be extracted by the lead author (GSB), and inputted into a customized electronic database. The customized electronic database will be based on the National Institute for Health and Care Excellence (NICE) evidence tables.[42] Data extracted will include authors, country, year of publication, title, *a priori* theme, study type, study design, body parts investigated (if applicable), study population(s), sample size, age of study participants, sex,

For peer review only - http://bmjopen.bmj.com/site/about/guidelines.xhtml

and standard-of-play. A second author (NKPP) will perform data extraction on 10% of studies, selected at random. Any discrepancies in data extraction between reviewers will result in the second reviewer (NKPP) extracting data from all studies. Following this, extracted data will be cross-checked for discrepancies, and any differences in data will be resolved between reviewers. Outcome data will be stratified into *a priori* themes of musculoskeletal health, general health, and wellbeing.

Stage 5: Collating, summarizing and reporting the results

Descriptive data and key findings will be collated and summarized for descriptive analysis and the results will be presented numerically and thematically. Individual specific study data and thematic data will be grouped into each *a priori* theme. Following the grouping of articles into each *a priori* theme, individual article data will be meta-aggregated to explore potential emergent themes.[41] Research gaps will be explored and tabulated through *a priori* theme and emergent theme collation. Specific article data will be tabulated and pertinent information will be aggregated into overall study data range for summarisation. Scoping review results will be presented in numeric and graphical representation for year of publication, geographic origin of publication, and *a priori* themes. A flow chart will be created to visually detail the screening and review process.[37] Emergent themes will be presented in tabular format, along with a narrative description of results.

Disseminating and Communicating Results

This scoping review is novel and will provide an overview of associations between cricket, health and wellbeing. Further, key research priorities relevant to stakeholders in cricket, including policymakers and sports governing bodies, will be clarified by this work.

For peer review only - http://bmjopen.bmj.com/site/about/guidelines.xhtml

Results will be published in a peer-reviewed sports and exercise medicine journal, with open access to increase information dissemination, regardless of positive or negative findings of the relationship between cricket participation, health, and wellbeing. In order to enhance knowledge translation of the findings, a multi-modal approach will be used for dissemination. Findings will be presented at conferences, multimedia resources will be (e.g. infographics, animations, videos, podcasts and blogs) will be created to disseminate via various social media platforms and through media release.

Conclusion

Scoping reviews are a scientifically validated method to answer broad research questions, and is the best methodology to understand the relationship between cricket participation, health and wellbeing, and summarise the knowledge gaps in this field. This scoping review will inform individuals and other stakeholders about the risk and benefits of cricket participation at all ages and standards-of-play. These findings may inform clinical decision making, policy changes, and future research agendas.

Author's Contributions

All authors were involved in planning, methodology design, and editing the manuscript. Garrett Bullock and Nirmala Panagodage Perera wrote the first draft of the manuscript. All authors approved this manuscript for submission.

Funding Statement

This study was funded by Centre for Sport, Exercise and Osteoarthritis Research Versus Arthritis (grant reference 21595).

For peer review only - http://bmjopen.bmj.com/site/about/guidelines.xhtml

References

1. International Cricket Council. ICC Members 2019 [cited 2019 4 March]. Available from: https://www.icc-cricket.com/about/members/about-our-members.
2. Network C. Record numbers playing cricket: CA. 2017. https://www.cricket.com.au/news/national-cricket-census-play-cricket-facilities-audit-james-sutherland-2016-17/2017-08-11.
3. Statista. Number of people participating in cricket in England from 2016 to 2018 2019 [Available from: https://www.statista.com/statistics/899199/cricket-participation-uk/ accessed May 6 2019.
4. Statista. India: What Sports Do You Regularly Participate In? 2019 [Available from: https://www.statista.com/statistics/562694/india-regular-participation-in-sports-by-type/ accessed May 7 2019.
5. Statistics ABO. Children's participation in cultural and leisure activities. *Canberra, Australian Bureau of Statistics* 2000
6. Maher C, Olds T, Dollman J. Adolescent sport in Australia: who, when, where and what? *ACHPER Australia Healthy Lifestyles Journal* 2009;56(1):11.
7. Bélanger M, Townsend N, Foster C. Age-related differences in physical activity profiles of English adults. *Preventive medicine* 2011;52(3-4):247-49.
8. Ainsworth BE, Haskell WL, Herrmann SD, et al. 2011 Compendium of Physical Activities: a second update of codes and MET values. *Medicine & science in sports & exercise* 2011;43(8):1575-81.
9. Warburton DE, Nicol CW, Bredin SS. Health benefits of physical activity: the evidence. *CMAJ : Canadian Medical Association journal = journal de l'Association medicale canadienne* 2006;174(6):801-9. doi: 10.1503/cmaj.051351 [published Online First: 2006/03/15]
10. Penedo FJ, Dahn JR. Exercise and well-being: a review of mental and physical health benefits associated with physical activity. *Current opinion in psychiatry* 2005;18(2):189-93.
11. Hallal PC, Andersen LB, Bull FC, et al. Global physical activity levels: surveillance progress, pitfalls, and prospects. *The lancet* 2012;380(9838):247-57.
12. Organization WH. Global action plan on physical activity 2018–2030: more active people for a healthier world: World Health Organization 2018.
13. Scheerder J, Thomis M, Vanreusel B, et al. Sports participation among females from adolescence to adulthood: A longitudinal study. *International review for the sociology of sport* 2006;41(3-4):413-30.
14. Schiphorst C, Murray A, Kelly P, et al. Infographic. Best investments for physical activity. *British journal of sports medicine* 2017;51(16):1227.
15. Noakes T, Durandt J. Physiological requirements of cricket. *Journal of Sports Sciences* 2000;18(12):919-29.
16. Johnstone JA, Ford PA. Physiologic profile of professional cricketers. *Journal of strength and conditioning research* 2010;24(11):2900-7. doi: 10.1519/JSC.0b013e3181bac3a7 [published Online First: 2010/10/27]
17. Eime RM, Young JA, Harvey JT, et al. A systematic review of the psychological and social benefits of participation in sport for children and adolescents: informing development of a conceptual model of health through sport. *The international*

For peer review only - http://bmjopen.bmj.com/site/about/guidelines.xhtml

journal of behavioral nutrition and physical activity 2013;10:98. doi: 10.1186/1479-5868-10-98 [published Online First: 2013/08/16]
18. Marlier M, Van Dyck D, Cardon G, et al. Interrelation of Sport Participation, Physical Activity, Social Capital and Mental Health in Disadvantaged Communities: A SEM-Analysis. *PloS one* 2015;10(10):e0140196. doi: 10.1371/journal.pone.0140196 [published Online First: 2015/10/10]
19. Jewett R, Sabiston CM, Brunet J, et al. School sport participation during adolescence and mental health in early adulthood. *The Journal of adolescent health : official publication of the Society for Adolescent Medicine* 2014;55(5):640-4. doi: 10.1016/j.jadohealth.2014.04.018 [published Online First: 2014/06/15]
20. Swann C, Telenta J, Draper G, et al. Youth sport as a context for supporting mental health: Adolescent male perspectives. *Psychology of sport and exercise* 2018;35:55-64.
21. Vella SA, Cliff DP, Magee CA, et al. Sports participation and parent-reported health-related quality of life in children: longitudinal associations. *The Journal of pediatrics* 2014;164(6):1469-74.
22. Soomro N, Strasiotto L, Sawdagar T, et al. Cricket Injury Epidemiology in the Twenty-First Century: What is the Burden? *Sports medicine (Auckland, NZ)* 2018;48(10):2301-16. doi: 10.1007/s40279-018-0960-y [published Online First: 2018/07/19]
23. Soomro N, Redrup D, Evens C, et al. Injury rate and patterns of Sydney grade cricketers: a prospective study of injuries in 408 cricketers. *Postgraduate medical journal* 2018;94(1114):425-31. doi: 10.1136/postgradmedj-2018-135861 [published Online First: 2018/07/28]
24. Dennis R, Farhart P, Goumas C, et al. Bowling workload and the risk of injury in elite cricket fast bowlers. *Journal of science and medicine in sport* 2003;6(3):359-67. [published Online First: 2003/11/12]
25. Orchard JW, Blanch P, Paoloni J, et al. Cricket fast bowling workload patterns as risk factors for tendon, muscle, bone and joint injuries. *British journal of sports medicine* 2015;49(16):1064-8. doi: 10.1136/bjsports-2014-093683 [published Online First: 2015/03/11]
26. Jones ME, Davies MA, Leyland KM, et al. The association of previous injury with joint pain, osteoarthritis, and joint replacement across all joints in former elite English cricketers. *Osteoarthritis and Cartilage* 2017;25 (Supplement 1):S200-S01.
27. Pardiwala DN, Rao NN, Varshney AV. Injuries in Cricket. *Sports health* 2018;10(3):217-22. doi: 10.1177/1941738117732318 [published Online First: 2017/10/04]
28. Jones ME, Davies MAM, Shah K, et al. The prevalence of hand and wrist osteoarthritis in elite former cricket and rugby union players. *Journal of science and medicine in sport* 2019 doi: 10.1016/j.jsams.2019.03.004 [published Online First: 2019/04/04]
29. Neil R, Bowles HCR, Fleming S, et al. The experience of competition stress and emotions in cricket. *Sport Psychologist* 2016;30(1):76-88. doi: 10.1123/tsp.2014-0077
30. Hundertmark J. Cricketers and mental health concerns. *Australasian psychiatry : bulletin of Royal Australian and New Zealand College of Psychiatrists* 2007;15(6):509-12. doi: 10.1080/10398560701458210 [published Online First: 2007/09/14]
31. Jones ME, Davies MAM, Leyland KM, et al. Osteoarthritis and other long-term health conditions in former elite cricketers. *Journal of science and medicine in sport* 2018;21(6):558-63. doi: 10.1016/j.jsams.2017.10.013 [published Online First: 2017/11/02]

For peer review only - http://bmjopen.bmj.com/site/about/guidelines.xhtml

32. Sahni M, Bhogal G. Anxiety, depression and perceived sporting performance among professional cricket players. *British journal of sports medicine* 2017 doi: 10.1136/bjsports-2017-097827.5 [published Online First: 2017/05/12]
33. Schuring N, Kerkhoffs G, Gray J, et al. The mental wellbeing of current and retired professional cricketers: an observational prospective cohort study. *The Physician and sportsmedicine* 2017;45(4):463-69. doi: 10.1080/00913847.2017.1386069 [published Online First: 2017/09/28]
34. Schuring N, Aoki H, Gray J, et al. Osteoarthritis is associated with symptoms of common mental disorders among former elite athletes. *Knee surgery, sports traumatology, arthroscopy : official journal of the ESSKA* 2017;25(10):3179-85. doi: 10.1007/s00167-016-4255-2 [published Online First: 2016/08/05]
35. Oja P, Titze S, Kokko S, et al. Health benefits of different sport disciplines for adults: systematic review of observational and intervention studies with meta-analysis. *Br J Sports Med* 2015;49(7):434-40.
36. Murray AD, Daines L, Archibald D, et al. The relationships between golf and health: a scoping review. *British journal of sports medicine* 2017;51(1):12-19. doi: 10.1136/bjsports-2016-096625 [published Online First: 2016/10/05]
37. Peters MD, Godfrey CM, Khalil H, et al. Guidance for conducting systematic scoping reviews. *International journal of evidence-based healthcare* 2015;13(3):141-46.
38. Murray A, Daines L, Archibald D, et al. The relationship and effects of golf on physical and mental health: a scoping review protocol. *Br J Sports Med* 2016;50(11):647-50.
39. Arksey H, O'Malley L. Scoping studies: towards a methodological framework. *International journal of social research methodology* 2005;8(1):19-32.
40. Levac D, Colquhoun H, O'Brien KK. Scoping studies: advancing the methodology. *Implementation science* 2010;5(1):69.
41. Munn Z, Tufanaru C, Aromataris E. JBI's systematic reviews: data extraction and synthesis. *AJN The American Journal of Nursing* 2014;114(7):49-54.
42. Conaghan PG, Dickson J, Grant RL. Care and management of osteoarthritis in adults: summary of NICE guidance. *Bmj* 2008;336(7642):502-03.

For peer review only - http://bmjopen.bmj.com/site/about/guidelines.xhtml

PRISMA 2009 Checklist

Section/topic	#	Checklist item	Reported on page #
**TITLE**			
Title	1	Identify the report as a systematic review, meta-analysis, or both.	1
**ABSTRACT**			
Structured summary	2	Provide a structured summary including, as applicable: background; objectives; data sources; study eligibility criteria, participants, and interventions; study appraisal and synthesis methods; results; limitations; conclusions and implications of key findings; systematic review registration number.	2
**INTRODUCTION**			
Rationale	3	Describe the rationale for the review in the context of what is already known.	4-5
Objectives	4	Provide an explicit statement of questions being addressed with reference to participants, interventions, comparisons, outcomes, and study design (PICOS).	5
**METHODS**			
Protocol and registration	5	Indicate if a review protocol exists, if and where it can be accessed (e.g., Web address), and, if available, provide registration information including registration number.	This is the review protocol attempting to be published
Eligibility criteria	6	Specify study characteristics (e.g., PICOS, length of follow-up) and report characteristics (e.g., years considered, language, publication status) used as criteria for eligibility, giving rationale.	7
Information sources	7	Describe all information sources (e.g., databases with dates of coverage, contact with study authors to identify additional studies) in the search and date last searched.	6-7
Search	8	Present full electronic search strategy for at least one database, including any limits used, such that it could be repeated.	6-7
Study selection	9	State the process for selecting studies (i.e., screening, eligibility, included in systematic review, and, if applicable, included in the meta-analysis).	8
Data collection process	10	Describe method of data extraction from reports (e.g., piloted forms, independently, in duplicate) and any processes for obtaining and confirming data from investigators.	8-9
Data items	11	List and define all variables for which data were sought (e.g., PICOS, funding sources) and any assumptions and simplifications made.	9

For peer review only

PRISMA 2009 Checklist

Risk of bias in individual studies	12	Describe methods used for assessing risk of bias of individual studies (including specification of whether this was done at the study or outcome level), and how this information is to be used in any data synthesis.	Scoping review not applicable
Summary measures	13	State the principal summary measures (e.g., risk ratio, difference in means).	9
Synthesis of results	14	Describe the methods of handling data and combining results of studies, if done, including measures of consistency (e.g., I^2) for each meta-analysis.	9

Section/topic	#	Checklist item	Reported on page #
Risk of bias across studies	15	Specify any assessment of risk of bias that may affect the cumulative evidence (e.g., publication bias, selective reporting within studies).	Not applicable scoping review
Additional analyses	16	Describe methods of additional analyses (e.g., sensitivity or subgroup analyses, meta-regression), if done, indicating which were pre-specified.	9
**RESULTS**			
Study selection	17	Give numbers of studies screened, assessed for eligibility, and included in the review, with reasons for exclusions at each stage, ideally with a flow diagram.	Not applicable as this is a protocol
Study characteristics	18	For each study, present characteristics for which data were extracted (e.g., study size, PICOS, follow-up period) and provide the citations.	
Risk of bias within studies	19	Present data on risk of bias of each study and, if available, any outcome level assessment (see item 12).	
Results of individual studies	20	For all outcomes considered (benefits or harms), present, for each study: (a) simple summary data for each intervention group (b) effect estimates and confidence intervals, ideally with a forest plot.	
Synthesis of results	21	Present results of each meta-analysis done, including confidence intervals and measures of consistency.	
Risk of bias across studies	22	Present results of any assessment of risk of bias across studies (see Item 15).	
Additional analysis	23	Give results of additional analyses, if done (e.g., sensitivity or subgroup analyses, meta-regression [see Item 16]).	
**DISCUSSION**			
Summary of evidence	24	Summarize the main findings including the strength of evidence for each main outcome; consider their relevance to key groups (e.g., healthcare providers, users, and policy makers).	

For peer review only

For peer review only - http://bmjopen.bmj.com/site/about/guidelines.xhtml

PRISMA 2009 Checklist

Limitations	25	Discuss limitations at study and outcome level (e.g., risk of bias), and at review-level (e.g., incomplete retrieval of identified research, reporting bias).	
Conclusions	26	Provide a general interpretation of the results in the context of other evidence, and implications for future research.	
**FUNDING**			
Funding	27	Describe sources of funding for the systematic review and other support (e.g., supply of data); role of funders for the systematic review.	3

From: Moher D, Liberati A, Tetzlaff J, Altman DG, The PRISMA Group (2009). Preferred Reporting Items for Systematic Reviews and Meta-Analyses: The PRISMA Statement. PLoS Med 6(7): e1000097. doi:10.1371/journal.pmed1000097

For more information, visit: www.prisma-statement.org.

Page 2 of 2

For peer review only

BMJ Open

The relationship between cricket participation, health, and wellbeing: A scoping review protocol

Journal:	*BMJ Open*
Manuscript ID	bmjopen-2019-032070.R1
Article Type:	Protocol
Date Submitted by the Author:	03-Sep-2019
Complete List of Authors:	Bullock, Garrett; University of Oxford, Nuffield Department of Orthopaedics, Rheumatology, and Musculoskeletal Sciences<br>Panagodage-Perera, Nirmala ; University of Oxford, Arthritis Research UK Centre for Sport, Exercise & Osteoarthritis, Nuffield Department of Orthopaedics, Rheumatology & Musculoskeletal Sciences<br>Murray, Andrew; University of Edinburgh, Physical Activity for Health Research Centre<br>Arden, Nigel; Nuffield Department of Orthopaedics, ;<br>Filbay, Stephanie; University of Oxford, Arthritis Research UK Centre for Sport, Exercise & Osteoarthritis, Nuffield Department of Orthopaedics, Rheumatology & Musculoskeletal Sciences
**Primary Subject Heading**:	Sports and exercise medicine
Secondary Subject Heading:	Mental health, Epidemiology
Keywords:	Physical Activity, Health Related Quality of Life, Injury, MENTAL HEALTH

SCHOLARONE™
Manuscripts

For peer review only - http://bmjopen.bmj.com/site/about/guidelines.xhtml

The relationship between cricket participation, health, and wellbeing: A scoping review protocol

Garrett S. Bullock PT, DPT,[1,2] Nirmala K. Panagodage Perera, MMed, PhD,[1,2,3,4] Andrew Murray, MD,[5] Nigel K. Arden, MD, FRCP,[1,2] Stephanie R. Filbay, B.Phty (Hons), PhD[1,2]

1. Centre for Sport, Exercise and Osteoarthritis Research Versus Arthritis; Oxford, United Kingdom
2. Nuffield Department of Orthopaedics, Rheumatology and Musculoskeletal Sciences, University of Oxford, Oxford, United Kingdom
3. Division of Physiotherapy, Department of Medical and Health Sciences, Linköping University, Sweden.
4. Latrobe Sports and Exercise Medicine Research Centre, College of Science, Health and Engineering, Latrobe University, Bundoora VIC 3086, Australia
5. Sport and Exercise, University of Edinburgh, Edinburgh, UK

Corresponding Author

Garrett S. Bullock PT, DPT
Nuffield Department of Orthopaedics, Rheumatology, and Musculoskeletal Sciences
University of Oxford
B4495
Oxford, United Kingdom
OX3 7LD
Telephone: (865) 227-374
Fax: (865) 768-876
garrett.bullock@wolfson.ox.ac.uk

Key Words: Physical Activity, Health Related Quality of Life, Injury, Mental Health, Musculoskeletal

Word Count: 2533

Competing Interests

All authors affirm that they have no involvement with any commercial organization that has a direct financial interest in any matter included in this manuscript.

For peer review only

Abstract

Introduction: Cricket is a popular sport played by 2.5 billion people of all ages and abilities. However, cricket participation is decreasing in the UK, despite an increased focus of governments on increasing sport participation to enhance public health. Understanding the health benefits, and mitigating the health risks of cricket participation may help cricket organisations promote cricket participation whilst optimising the long-term health of cricket participants. Currently there is no literature review on the relationship between cricket participation, health, and wellbeing; thus, this relationship remains unclear. Therefore, the aim of this scoping review is to i) investigate the relationship between cricket participation, health and wellbeing and ii) identify the research gaps related to cricket, health and wellbeing

Methods and analysis: Due to the broad nature of our research question, the large number of health outcomes assessed within the cricket literature, and to facilitate identification of research gaps, a scoping review methodology was utilised. The methodology of this paper was informed by previous scoping review protocols and best practice methodological frameworks. Medline, CINAHL, Embase, Scopus, PsycINFO, SPORTDiscus, Cochrane Library, EBSCO, Web of Science and PEDro, and grey literature sources (Google Scholar, Clinicaltrials.gov, ISRCTN Registry and Proquest) will be systematically searched. Studies that assess a construct related to health and/or wellbeing in current and/or former cricketers from all ages and standards-of-play will be eligible. Two reviewers will independently screen full-texts of identified studies for eligibility, and perform data extraction. Results will be presented in tabular and graphical form and reported descriptively.

Ethics and dissemination: This research is exempt from ethics approval due to the data being available through published and public available resources. Results will be published in a peer-reviewed sports and exercise medicine journal regardless of positive or negative findings. In addition, results will be disseminated through multiple platforms including

For peer review only - http://bmjopen.bmj.com/site/about/guidelines.xhtml

conference presentations and social media using multimedia resources (e.g. infographics, animations, videos, podcasts, and blogs) to engage stakeholder groups including cricketers, cricket coaches, sporting bodies, sports medicine professionals and policy makers. There findings will inform clinical decision making, policy changes, and future research agendas.

Key Words: Physical Activity, Health Related Quality of Life, Injury, Mental Health, Musculoskeletal

Strengths and Limitations

- Scoping reviews are a scientifically validated method to answer broad research questions, and is the best methodology to provide an overview of all literature investigating the relationship between cricket participation, health and wellbeing.
- This scoping review will include grey literature to increase the scope and breadth of the review and screening process.
- Individual article data will be meta-aggregated to explore emergent themes, with potential to provide new insights and inform future research
- Specific articles cannot be analyzed for methodological risk of bias, decreasing the interpretability of the results.

For peer review only

Introduction

Cricket is a popular sport with approximately 2.5 billion people of all ages and abilities participating.[1] Cricket is played by 1.4 million people in Australia,[2] nearly 300,000 people in the United Kingdom,[3] and over 5 million people in India.[4] Further, cricket is popular among youth,[5 6] many of whom continue to play cricket into adulthood.[7] Cricket has also become increasingly popular among females with more than 27% of all Australian cricketers being female.[2] Cricket is played with 11 individuals per team, over 5 days (test cricket), one day (50 overs) or over four hours (Twenty20). The 2011 Compendium of Physical Activities list cricket as a sport that can provide moderate-intensity physical activity.[8] Regular physical activity is an important determinant of general health, life expectancy[9] and overall wellbeing.[10]

Over 31% of all adults worldwide are physically inactive, with physical inactivity levels ranging from 17% in Asia, to 43% in North America.[11] To counteract inactivity, sports participation is promoted.[12] Sport participation provides opportunities to be physically active across the lifespan.[13 14] Cricket participation can improve fitness[15 16] and strength,[16] and has psychological benefits for participants[17-19] including improved self-esteem, social connections and overall wellbeing.[17] Mental health and health-related quality of life (HRQoL) is higher in cricketers compared to the general population.[20 21] However, cricket participation is associated with injury[22-25] which can result in persistent joint pain and post-traumatic osteoarthritis.[26] Specifically, injury incidences has been reported to be up to 53 injuries per 10,000 athlete exposures,[27] with former cricketer reporting greater osteoarthritis compared to former rugby players.[28] Further, some cricketers experience increased levels of stress[29 30] and depression,[31 32] which can negatively impact HRQoL,[26 33 34] Thus, cricket participation may have both positive and negative impacts on health and wellbeing. Due to

For peer review only - http://bmjopen.bmj.com/site/about/guidelines.xhtml

the high rate of global physical inactivity,[11] the worldwide popularity of cricket,[2-4] and its viability as an outlet for physical activity across the lifespan,[13 14] information regarding the potential risks and benefits of participation in specific sports is needed to enable informed decision making for participants.

The link between sport participation and beneficial health outcomes have been synthesised in previous systematic reviews for golf, cycling, and sport and dance.[35-37] These studies found that participation in these activities had a positive relationship with physical health and wellbeing.[35-37] However, the relationship between cricket, health and wellbeing has not been investigated. Thus, there is a need to map the current evidence related to cricket, health and wellbeing, and identify key research priorities. This overview would also enable key stakeholders (including cricket participants, health professionals and sporting bodies) to make evidence informed decisions relating to cricket participation. Specifically, these data will inform stakeholders on the health and wellbeing risks and benefits of cricket participation in order to make individual and organizational decisions on the viability of promoting cricket participation as a health enhancing form of physical activity at different standards-of-play and for different age-groups. Further, identifying the gaps in the literature will allow specific cricket related research to be initiated to improve cricket participant health and wellbeing. Therefore, the aim of this scoping review is to (1) investigate the relationship between cricket participation, health and wellbeing at all ages and standards-of-play; (2) identify research gaps in the existing literature on cricket, health and wellbeing.

Methods

Stakeholder Involvement

For peer review only - http://bmjopen.bmj.com/site/about/guidelines.xhtml

Findings from two qualitative studies investigating the relationship of physical activity and quality of life in former elite cricketers,[38 39] highlighted a need for further research investigating the relationship between cricket participation and health. It was determined that a scoping review would provide valuable information regarding the relationship between cricket participation, health and wellbeing, whilst identifying key knowledge gaps to guide future research agendas. An international stakeholder group will be established, comprising current and former cricketers, cricket coaches, sports medicine professionals, cricket-related researchers and representatives from cricket sporting bodies. This key stakeholder group will meet virtually, to discuss preliminary results and interpretation of findings, review a draft of the manuscript, and provide input into the plan for dissemination of research findings.

Patient and Public Involvement

No patients were involved.

Study Design

The purpose of a scoping review is to describe all available evidence underpinning a given research question drawing upon research from all possible sources, consequently scoping reviews are broad in nature. This is in comparison to a systematic review which can only investigate one specific topic; as a result, a scoping review methodology was commenced.[40 41] The framework adopted for this scoping review follows existing best practice methodology.[40-44] The methodology was guided by the recommended five-stage process: identify the research question; identify relevant studies; select articles using *a priori* inclusion/exclusion criteria; chart data; collate, summarize, and report results.[40-42] The proposed study is planned to be commenced during September 2019, and is estimated to

For peer review only - http://bmjopen.bmj.com/site/about/guidelines.xhtml

conclude in March 2020. This research is exempt from ethical approval since it is a review of previously published articles.

Stage 1: Identify the research question

The general research question was developed through exploration of the literature, multi-disciplinary group discussions and collaborating with experts in cricket. To reflect the context, content and the population included in the review,[40 41] the following broad research question was proposed: *what is known about the relationship between cricket participation, health and wellbeing?*

Stage 2: Identify relevant studies

A preliminary search to identify key words and index terms

A preliminary search was conducted on the major clinical and grey literature databases.[40-42] Databases included Medline, Google Scholar, and ProQuest Dissertations and Theses Global. Consistent with previous studies[42] exploratory search terms were kept broad, to be as inclusive as possible. Search terms included "*cricket*," "*health*," and "*review*." The exploratory search found 37 articles in Medline. The first 200 articles in Google Scholar were searched. Twenty-eight articles were identified as pertinent from Medline and Google Scholar. No relevant articles were found in ProQuest. These 28 articles references were then searched for further relevant articles.

The titles and abstracts of these 28 articles were then analysed for relevant search terms. The preliminary search identified a large number of irrelevant studies involving cricket insects, cadaveric, or in vitro investigations, consequently the search terms were updated to exclude articles with cadaver* or "in situ" or "in vitro" or "insects" in the title and/or abstract. The

For peer review only - http://bmjopen.bmj.com/site/about/guidelines.xhtml

final search strategy was created to keep the search broad for greatest inclusion, while excluding specific irrelevant studies identified through the preliminary search. A medical librarian assisted by ensuring the search syntax was appropriate for each database.

Search strategy

Ten databases (Medline, CINAHL, Embase, Scopus, PsycINFO, SPORTDiscus, Cochrane Library, EBSCO, Web of Science and PEDro) will be electronically searched. Google Scholar, Clinicaltrials.gov, ISRCTN Registry and Proquest Dissertation and Theses will be searched for grey literature. The search strategy will be as follows, *"cricket** NOT (*cadaver* or "in situ" or "in vitro" or animals or insects*)*"* (Appendix 1) . Articles will be tracked in EndNote® X9 (Clarivate Analytics, 2018).

Study eligibility criteria

Please refer to Table 1 for inclusion and exclusion criteria.

Table 1. Inclusion and exclusion criteria

Inclusion Criteria	Exclusion Criteria
• Assesses a construct related to health (e.g. injury, pain, physiological function, physical activity, tobacco use, alcohol use, body mass index, nutrition, diabetes, cardiovascular disease) and/or wellbeing (e.g. mental health, depression, mood, anxiety, health related quality of life, resilience) in current and/or former cricketers (of any age, sex or competition level)	• Cricket performance parameters (e.g. bowling speed, wins and losses, and bowling average)
• Primary research studies, reviews, meta-analyses, guidelines or grey literature (including unpublished and ongoing trials, annual reports, dissertations and conference abstracts)	• Biomechanics (force, torque, kinematics and electromyography), joint range of motion/flexibility

For peer review only - http://bmjopen.bmj.com/site/about/guidelines.xhtml

• Human studies	• Cadaveric, or in situ models studies
• Articles published in English	• Editorials, periodicals, letters to the editor

Stage 3: Study selection

Titles and abstracts will be screened by the lead author (GSB) for eligibility, and full-text articles will be retrieved and screened by the same author (GSB) against the inclusion/exclusion criteria. A second author (NKPP) will complete the same screening process on a random sample of 10% of the articles.[42] Any title and abstract screening disputes will be resolved through the consensus of the two authors. If concordance is less than 90%, the full title and abstract screening will be performed by the second author (NKPP). Following title and abstract screening, the full text of all potentially eligible articles will be retrieved. Firstly, we will attempt to access articles through university online library portals. The online library portals will be available through collaborating institutions in the United Kingdom, Sweden, Australia, and the United States. If the article cannot be retrieved through the university online library portals, the authors will be contacted to request full-text, and if required inter library loan with the assistance of a librarian will be attempted. If a full-text article cannot be retrieved following consultation with a librarian, it will be excluded from the review.[42] If there are any discrepancies following full-text screening, a third author (SRF) will arbitrate all disputes, and decide on final article inclusion.

Stage 4: Data extraction

Data extraction procedures with follow best systematic review practice guidelines.[45] Data will be extracted by the lead author (GSB), and inputted into a customized electronic database. The customized electronic database will be based on the National Institute for

For peer review only - http://bmjopen.bmj.com/site/about/guidelines.xhtml

Health and Care Excellence (NICE) evidence tables.[46] Quantitative data that will be extracted will include publication year, study type (primary, secondary, or grey literature), country of origin, age group, competition level, study design, study description, surgical procedure (if applicable), analysis design, and key findings. Qualitative data will be extracted through qualitative synthesis of related topics.[45] A second author (NKPP) will perform data extraction on 10% of studies, selected at random. Any discrepancies in data extraction between reviewers will result in the second reviewer (NKPP) extracting data from all studies. Following this, extracted data will be cross-checked for discrepancies, and any differences in data will be resolved between reviewers. Outcome data will be stratified into *a priori* themes of musculoskeletal health, general health, and wellbeing.

Stage 5: Collating, summarizing and reporting the results

Descriptive data and key findings will be collated and summarized for descriptive analysis and the results will be presented numerically and thematically. Individual specific study quantitative and qualitative data and thematic data will be grouped into each *a priori* theme. Following the grouping of articles into each *a priori* theme, individual article data will be meta-aggregated to explore potential emergent themes.[45] Specifically, quantitative data will be extracted, sorted into relevant themes (e.g. musculoskeletal health, mental health, physiological health), and descriptively reported.[45] Qualitative data will be synthesized in excel, through a six stage process. This six stage process includes becoming familiar with the data, generating initial codes, searching for themes, reviewing themes, defining themes, and writing up. [47 48]Research gaps will be explored and tabulated through *a priori* theme and emergent theme collation. Specific article data will be tabulated and pertinent information will be aggregated into overall study data range for summarisation. Scoping review results will be presented in numeric and graphical representation for year of publication, geographic

For peer review only - http://bmjopen.bmj.com/site/about/guidelines.xhtml

origin of publication, and *a priori* themes. A flow chart will be created to visually detail the screening and review process.[43] Emergent themes will be presented in tabular format, along with a narrative description of results.

Disseminating and Communicating Results

This scoping review of cricket health and wellbeing is novel and will provide an overview of associations between cricket, health and wellbeing. Further, key research priorities relevant to stakeholders in cricket, including policymakers and sports governing bodies, will be clarified by this work.

Results will be published in a peer-reviewed sports and exercise medicine journal, with open access to increase information dissemination, regardless of positive or negative findings of the relationship between cricket participation, health, and wellbeing. In order to enhance knowledge translation of the findings, a multi-modal approach will be used for dissemination. Findings will be presented at conferences, multimedia resources will be (e.g. infographics, animations, videos, podcasts and blogs) will be created to disseminate findings via various social media platforms and through media release.

Conclusion

The aims and methodological study design were created in concordance with cricket stakeholders (current and former cricketers, physicians, physiotherapists, and governing bodies) in order to have a greater understanding of the relationship between cricket participation, health and wellbeing. Scoping reviews are a scientifically validated method to answer broad research questions, and summarise the knowledge gaps in this field. This scoping review will inform individuals and other stakeholders about the risk and benefits of

For peer review only - http://bmjopen.bmj.com/site/about/guidelines.xhtml

cricket participation at all ages and standards-of-play. These findings may inform clinical decision making, policy changes, and future research agendas.

Author's Contributions

GSB, NPP, NA, and SRF conceived the study idea. GSB, NPP, AM, NA, and SRF were involved in methodological design and planning. GSB, NPP, and SRF wrote the first draft of the manuscript. GSB, NPP, AM, NA, and SRF all critically revised the manuscript. GSB, NPP, AM, NA, and SRF all approved the final version of the manuscript.

Funding Statement

This study was funded by Centre for Sport, Exercise and Osteoarthritis Research *Versus Arthritis* (grant reference 21595). The Centre for Sport, Exercise and Osteoarthritis Research *Versus Arthritis* had no role in study design, data collection, data analysis and interpretation, manuscript preparation, nor in the decision to submit the paper for publication.

For peer review only

References

1. International Cricket Council. ICC Members 2019 [cited 2019 4 March]. Available from: https://www.icc-cricket.com/about/members/about-our-members.
2. Network C. Record numbers playing cricket: CA. 2017. https://www.cricket.com.au/news/national-cricket-census-play-cricket-facilities-audit-james-sutherland-2016-17/2017-08-11.
3. Statista. Number of people participating in cricket in England from 2016 to 2018 2019 [Available from: https://www.statista.com/statistics/899199/cricket-participation-uk/ accessed May 6 2019.
4. Statista. India: What Sports Do You Regularly Participate In? 2019 [Available from: https://www.statista.com/statistics/562694/india-regular-participation-in-sports-by-type/ accessed May 7 2019.
5. Statistics ABO. Children's participation in cultural and leisure activities. *Canberra, Australian Bureau of Statistics* 2000
6. Maher C, Olds T, Dollman J. Adolescent sport in Australia: who, when, where and what? *ACHPER Australia Healthy Lifestyles Journal* 2009;56(1):11.
7. Bélanger M, Townsend N, Foster C. Age-related differences in physical activity profiles of English adults. *Preventive medicine* 2011;52(3-4):247-49.
8. Ainsworth BE, Haskell WL, Herrmann SD, et al. 2011 Compendium of Physical Activities: a second update of codes and MET values. *Medicine & science in sports & exercise* 2011;43(8):1575-81.
9. Warburton DE, Nicol CW, Bredin SS. Health benefits of physical activity: the evidence. *CMAJ : Canadian Medical Association journal = journal de l'Association medicale canadienne* 2006;174(6):801-9. doi: 10.1503/cmaj.051351 [published Online First: 2006/03/15]
10. Penedo FJ, Dahn JR. Exercise and well-being: a review of mental and physical health benefits associated with physical activity. *Current opinion in psychiatry* 2005;18(2):189-93.
11. Hallal PC, Andersen LB, Bull FC, et al. Global physical activity levels: surveillance progress, pitfalls, and prospects. *The lancet* 2012;380(9838):247-57.
12. Organization WH. Global action plan on physical activity 2018–2030: more active people for a healthier world: World Health Organization 2018.
13. Scheerder J, Thomis M, Vanreusel B, et al. Sports participation among females from adolescence to adulthood: A longitudinal study. *International review for the sociology of sport* 2006;41(3-4):413-30.
14. Schiphorst C, Murray A, Kelly P, et al. Infographic. Best investments for physical activity. *British journal of sports medicine* 2017;51(16):1227.
15. Noakes T, Durandt J. Physiological requirements of cricket. *Journal of Sports Sciences* 2000;18(12):919-29.
16. Johnstone JA, Ford PA. Physiologic profile of professional cricketers. *Journal of strength and conditioning research* 2010;24(11):2900-7. doi: 10.1519/JSC.0b013e3181bac3a7 [published Online First: 2010/10/27]
17. Eime RM, Young JA, Harvey JT, et al. A systematic review of the psychological and social benefits of participation in sport for children and adolescents: informing development of a conceptual model of health through sport. *The international*

For peer review only - http://bmjopen.bmj.com/site/about/guidelines.xhtml

journal of behavioral nutrition and physical activity 2013;10:98. doi: 10.1186/1479-5868-10-98 [published Online First: 2013/08/16]

18. Marlier M, Van Dyck D, Cardon G, et al. Interrelation of Sport Participation, Physical Activity, Social Capital and Mental Health in Disadvantaged Communities: A SEM-Analysis. *PloS one* 2015;10(10):e0140196. doi: 10.1371/journal.pone.0140196 [published Online First: 2015/10/10]
19. Jewett R, Sabiston CM, Brunet J, et al. School sport participation during adolescence and mental health in early adulthood. *The Journal of adolescent health : official publication of the Society for Adolescent Medicine* 2014;55(5):640-4. doi: 10.1016/j.jadohealth.2014.04.018 [published Online First: 2014/06/15]
20. Swann C, Telenta J, Draper G, et al. Youth sport as a context for supporting mental health: Adolescent male perspectives. *Psychology of sport and exercise* 2018;35:55-64.
21. Vella SA, Cliff DP, Magee CA, et al. Sports participation and parent-reported health-related quality of life in children: longitudinal associations. *The Journal of pediatrics* 2014;164(6):1469-74.
22. Soomro N, Strasiotto L, Sawdagar T, et al. Cricket Injury Epidemiology in the Twenty-First Century: What is the Burden? *Sports medicine (Auckland, NZ)* 2018;48(10):2301-16. doi: 10.1007/s40279-018-0960-y [published Online First: 2018/07/19]
23. Soomro N, Redrup D, Evens C, et al. Injury rate and patterns of Sydney grade cricketers: a prospective study of injuries in 408 cricketers. *Postgraduate medical journal* 2018;94(1114):425-31. doi: 10.1136/postgradmedj-2018-135861 [published Online First: 2018/07/28]
24. Dennis R, Farhart P, Goumas C, et al. Bowling workload and the risk of injury in elite cricket fast bowlers. *Journal of science and medicine in sport* 2003;6(3):359-67. [published Online First: 2003/11/12]
25. Orchard JW, Blanch P, Paoloni J, et al. Cricket fast bowling workload patterns as risk factors for tendon, muscle, bone and joint injuries. *British journal of sports medicine* 2015;49(16):1064-8. doi: 10.1136/bjsports-2014-093683 [published Online First: 2015/03/11]
26. Jones ME, Davies MA, Leyland KM, et al. The association of previous injury with joint pain, osteoarthritis, and joint replacement across all joints in former elite English cricketers. *Osteoarthritis and Cartilage* 2017;25 (Supplement 1):S200-S01.
27. Pardiwala DN, Rao NN, Varshney AV. Injuries in Cricket. *Sports health* 2018;10(3):217-22. doi: 10.1177/1941738117732318 [published Online First: 2017/10/04]
28. Jones ME, Davies MAM, Shah K, et al. The prevalence of hand and wrist osteoarthritis in elite former cricket and rugby union players. *Journal of science and medicine in sport* 2019 doi: 10.1016/j.jsams.2019.03.004 [published Online First: 2019/04/04]
29. Neil R, Bowles HCR, Fleming S, et al. The Experience of Competition Stress and Emotions in Cricket. *Sport Psychologist* 2016;30(1):76-88. doi: 10.1123/tsp.2014-0077
30. Hundertmark J. Cricketers and mental health concerns. *Australasian psychiatry : bulletin of Royal Australian and New Zealand College of Psychiatrists* 2007;15(6):509-12. doi: 10.1080/10398560701458210 [published Online First: 2007/09/14]
31. Jones ME, Davies MAM, Leyland KM, et al. Osteoarthritis and other long-term health conditions in former elite cricketers. *Journal of science and medicine in sport* 2018;21(6):558-63. doi: 10.1016/j.jsams.2017.10.013 [published Online First: 2017/11/02]

For peer review only - http://bmjopen.bmj.com/site/about/guidelines.xhtml

32. Sahni M, Bhogal G. Anxiety, depression and perceived sporting performance among professional cricket players. *British journal of sports medicine* 2017 doi: 10.1136/bjsports-2017-097827.5 [published Online First: 2017/05/12]
33. Schuring N, Kerkhoffs G, Gray J, et al. The mental wellbeing of current and retired professional cricketers: an observational prospective cohort study. *The Physician and sportsmedicine* 2017;45(4):463-69. doi: 10.1080/00913847.2017.1386069 [published Online First: 2017/09/28]
34. Schuring N, Aoki H, Gray J, et al. Osteoarthritis is associated with symptoms of common mental disorders among former elite athletes. *Knee surgery, sports traumatology, arthroscopy : official journal of the ESSKA* 2017;25(10):3179-85. doi: 10.1007/s00167-016-4255-2 [published Online First: 2016/08/05]
35. Oja P, Titze S, Kokko S, et al. Health benefits of different sport disciplines for adults: systematic review of observational and intervention studies with meta-analysis. *Br J Sports Med* 2015;49(7):434-40.
36. Oja P, Titze S, Bauman A, et al. Health benefits of cycling: a systematic review. *Scandinavian journal of medicine & science in sports* 2011;21(4):496-509.
37. Mansfield L, Kay T, Meads C, et al. Sport and dance interventions for healthy young people (15–24 years) to promote subjective well-being: a systematic review. *BMJ open* 2018;8(7):e020959.
38. Filbay SR, Bishop F, Peirce N, et al. Common attributes in retired professional cricketers that may enhance or hinder quality of life after retirement: a qualitative study. *BMJ open* 2017;7(7):e016541. doi: 10.1136/bmjopen-2017-016541 [published Online First: 2017/07/29]
39. Filbay SR, Bishop FL, Peirce N, et al. Physical activity in former elite cricketers and strategies for promoting physical activity after retirement from cricket: a qualitative study. *BMJ open* 2017;7(11):e017785. doi: 10.1136/bmjopen-2017-017785 [published Online First: 2017/11/21]
40. Arksey H, O'Malley L. Scoping studies: towards a methodological framework. *International journal of social research methodology* 2005;8(1):19-32.
41. Levac D, Colquhoun H, O'Brien KK. Scoping studies: advancing the methodology. *Implementation science* 2010;5(1):69.
42. Murray AD, Daines L, Archibald D, et al. The relationships between golf and health: a scoping review. *British journal of sports medicine* 2017;51(1):12-19. doi: 10.1136/bjsports-2016-096625 [published Online First: 2016/10/05]
43. Peters MD, Godfrey CM, Khalil H, et al. Guidance for conducting systematic scoping reviews. *International journal of evidence-based healthcare* 2015;13(3):141-46.
44. Murray A, Daines L, Archibald D, et al. The relationship and effects of golf on physical and mental health: a scoping review protocol. *Br J Sports Med* 2016;50(11):647-50.
45. Munn Z, Tufanaru C, Aromataris E. JBI's systematic reviews: data extraction and synthesis. *AJN The American Journal of Nursing* 2014;114(7):49-54.
46. Conaghan PG, Dickson J, Grant RL. Care and management of osteoarthritis in adults: summary of NICE guidance. *Bmj* 2008;336(7642):502-03.
47. Bree RT, Gallagher G. Using Microsoft Excel to code and thematically analyse qualitative data: a simple, cost-effective approach. *AISHE-J: The All Ireland Journal of Teaching and Learning in Higher Education* 2016;8(2)

For peer review only - http://bmjopen.bmj.com/site/about/guidelines.xhtml

48. Maguire M, Delahunt B. Doing a thematic analysis: A practical, step-by-step guide for learning and teaching scholars. *AISHE-J: The All Ireland Journal of Teaching and Learning in Higher Education* 2017;9(3)

For peer review only

For peer review only - http://bmjopen.bmj.com/site/about/guidelines.xhtml

Appendix ##: Example of full search strategy.

Medline and PubMed:
*cricket** NOT (*cadaver* or "in situ" or "in vitro" or animals or insects*)

For peer review only

Preferred Reporting Items for Systematic reviews and Meta-Analyses extension for Scoping Reviews (PRISMA-ScR) Checklist

SECTION	ITEM	PRISMA-ScR CHECKLIST ITEM	REPORTED ON PAGE #
**TITLE**			
Title	1	Identify the report as a scoping review.	1
**ABSTRACT**			
Structured summary	2	Provide a structured summary that includes (as applicable): background, objectives, eligibility criteria, sources of evidence, charting methods, results, and conclusions that relate to the review questions and objectives.	2
**INTRODUCTION**			
Rationale	3	Describe the rationale for the review in the context of what is already known. Explain why the review questions/objectives lend themselves to a scoping review approach.	4-5
Objectives	4	Provide an explicit statement of the questions and objectives being addressed with reference to their key elements (e.g., population or participants, concepts, and context) or other relevant key elements used to conceptualize the review questions and/or objectives.	5
**METHODS**			
Protocol and registration	5	Indicate whether a review protocol exists; state if and where it can be accessed (e.g., a Web address); and if available, provide registration information, including the registration number.	This is the prot ocol
Eligibility criteria	6	Specify characteristics of the sources of evidence used as eligibility criteria (e.g., years considered, language, and publication status), and provide a rationale.	7-8
Information sources*	7	Describe all information sources in the search (e.g., databases with dates of coverage and contact with authors to identify additional sources), as well as the date the most recent search was executed.	6-7
Search	8	Present the full electronic search strategy for at least 1 database, including any limits used, such that it could be repeated.	6-7
Selection of sources of evidence†	9	State the process for selecting sources of evidence (i.e., screening and eligibility) included in the scoping review.	8
Data charting process‡	10	Describe the methods of charting data from the included sources of evidence (e.g., calibrated forms or forms that have been tested by the team before their use, and whether data charting was done independently or in duplicate) and any processes for obtaining and confirming data from investigators.	8-9
Data items	11	List and define all variables for which data were sought and any assumptions and simplifications made.	9
Critical appraisal of individual sources of evidence§	12	If done, provide a rationale for conducting a critical appraisal of included sources of evidence; describe the methods used and how this information was used in any data synthesis (if appropriate).	Only study met hodology will b e appraised
Synthesis of results	13	Describe the methods of handling and summarizing the data that were charted.	9

St. Michael's
Inspired Care. Inspiring Science.

For peer review only - http://bmjopen.bmj.com/site/about/guidelines.xhtml

SECTION	ITEM	PRISMA-ScR CHECKLIST ITEM	REPORTED ON PAGE #
**RESULTS**			
Selection of sources of evidence	14	Give numbers of sources of evidence screened, assessed for eligibility, and included in the review, with reasons for exclusions at each stage, ideally using a flow diagram.	Protocol
Characteristics of sources of evidence	15	For each source of evidence, present characteristics for which data were charted and provide the citations.	Protocol
Critical appraisal within sources of evidence	16	If done, present data on critical appraisal of included sources of evidence (see item 12).	Protocol
Results of individual sources of evidence	17	For each included source of evidence, present the relevant data that were charted that relate to the review questions and objectives.	Protocol
Synthesis of results	18	Summarize and/or present the charting results as they relate to the review questions and objectives.	Protocol
**DISCUSSION**			
Summary of evidence	19	Summarize the main results (including an overview of concepts, themes, and types of evidence available), link to the review questions and objectives, and consider the relevance to key groups.	Protocol
Limitations	20	Discuss the limitations of the scoping review process.	Protocol
Conclusions	21	Provide a general interpretation of the results with respect to the review questions and objectives, as well as potential implications and/or next steps.	Protocol
**FUNDING**			
Funding	22	Describe sources of funding for the included sources of evidence, as well as sources of funding for the scoping review. Describe the role of the funders of the scoping review.	12

JBI = Joanna Briggs Institute; PRISMA-ScR = Preferred Reporting Items for Systematic reviews and Meta-Analyses extension for Scoping Reviews.
* Where *sources of evidence* (see second footnote) are compiled from, such as bibliographic databases, social media platforms, and Web sites.
† A more inclusive/heterogeneous term used to account for the different types of evidence or data sources (e.g., quantitative and/or qualitative research, expert opinion, and policy documents) that may be eligible in a scoping review as opposed to only studies. This is not to be confused with *information sources* (see first footnote).
‡ The frameworks by Arksey and O'Malley (6) and Levac and colleagues (7) and the JBI guidance (4, 5) refer to the process of data extraction in a scoping review as data charting.
§ The process of systematically examining research evidence to assess its validity, results, and relevance before using it to inform a decision. This term is used for items 12 and 19 instead of "risk of bias" (which is more applicable to systematic reviews of interventions) to include and acknowledge the various sources of evidence that may be used in a scoping review (e.g., quantitative and/or qualitative research, expert opinion, and policy document).

From: Tricco AC, Lillie E, Zarin W, O'Brien KK, Colquhoun H, Levac D, et al. PRISMA Extension for Scoping Reviews (PRISMA-ScR): Checklist and Explanation. Ann Intern Med. ;169:467–473. doi: 10.7326/M18-0850

For peer review only - http://bmjopen.bmj.com/site/about/guidelines.xhtml

BMJ Open

The relationship between cricket participation, health, and wellbeing: A scoping review protocol

Journal:	*BMJ Open*
Manuscript ID	bmjopen-2019-032070.R2
Article Type:	Protocol
Date Submitted by the Author:	04-Oct-2019
Complete List of Authors:	Bullock, Garrett; University of Oxford, Nuffield Department of Orthopaedics, Rheumatology, and Musculoskeletal Sciences<br>Panagodage-Perera, Nirmala ; University of Oxford, Arthritis Research UK Centre for Sport, Exercise & Osteoarthritis, Nuffield Department of Orthopaedics, Rheumatology & Musculoskeletal Sciences<br>Murray, Andrew; University of Edinburgh, Physical Activity for Health Research Centre<br>Arden, Nigel; Nuffield Department of Orthopaedics, ;<br>Filbay, Stephanie; University of Oxford, Arthritis Research UK Centre for Sport, Exercise & Osteoarthritis, Nuffield Department of Orthopaedics, Rheumatology & Musculoskeletal Sciences
**Primary Subject Heading**:	Sports and exercise medicine
Secondary Subject Heading:	Mental health, Epidemiology
Keywords:	Physical Activity, Health Related Quality of Life, Injury, MENTAL HEALTH

SCHOLARONE™
Manuscripts

The relationship between cricket participation, health, and wellbeing: A scoping review protocol

Garrett S. Bullock PT, DPT,[1,2] Nirmala K. Panagodage Perera, MMed, PhD,[1,2,3,4] Andrew Murray, MD,[5] Nigel K. Arden, MD, FRCP,[1,2] Stephanie R. Filbay, B.Phty (Hons), PhD[1,2]

1. Centre for Sport, Exercise and Osteoarthritis Research Versus Arthritis; Oxford, United Kingdom
2. Nuffield Department of Orthopaedics, Rheumatology and Musculoskeletal Sciences, University of Oxford, Oxford, United Kingdom
3. Division of Physiotherapy, Department of Medical and Health Sciences, Linköping University, Sweden.
4. Latrobe Sports and Exercise Medicine Research Centre, College of Science, Health and Engineering, Latrobe University, Bundoora VIC 3086, Australia
5. Sport and Exercise, University of Edinburgh, Edinburgh, UK

Corresponding Author

Garrett S. Bullock PT, DPT
Nuffield Department of Orthopaedics, Rheumatology, and Musculoskeletal Sciences
University of Oxford
B4495
Oxford, United Kingdom
OX3 7LD
Telephone: (865) 227-374
Fax: (865) 768-876
garrett.bullock@wolfson.ox.ac.uk

For peer review only

Key Words: Physical Activity, Health Related Quality of Life, Injury, Mental Health, Musculoskeletal

Word Count: 2533

Competing Interests

All authors affirm that they have no involvement with any commercial organization that has a direct financial interest in any matter included in this manuscript.

For peer review only - http://bmjopen.bmj.com/site/about/guidelines.xhtml

Abstract

Introduction: Cricket is a popular sport played by 2.5 billion people of all ages and abilities. However, cricket participation is decreasing in the UK, despite an increased focus of governments on increasing sport participation to enhance public health. Understanding the health benefits, and mitigating the health risks of cricket participation may help cricket organisations promote cricket participation whilst optimising the long-term health of cricket participants. Currently there is no literature review on the relationship between cricket participation, health, and wellbeing; thus, this relationship remains unclear. Therefore, the aim of this scoping review is to i) investigate the relationship between cricket participation, health and wellbeing and ii) identify the research gaps related to cricket, health and wellbeing

Methods and analysis: Due to the broad nature of our research question, the large number of health outcomes assessed within the cricket literature, and to facilitate identification of research gaps, a scoping review methodology was utilised. The methodology of this paper was informed by previous scoping review protocols and best practice methodological frameworks. Medline, CINAHL, Embase, Scopus, PsycINFO, SPORTDiscus, Cochrane Library, EBSCO, Web of Science and PEDro, and grey literature sources (Google Scholar, Clinicaltrials.gov, ISRCTN Registry and Proquest) will be systematically searched. Studies that assess a construct related to health and/or wellbeing in current and/or former cricketers from all ages and standards-of-play will be eligible. Two reviewers will independently screen full-texts of identified studies for eligibility, and perform data extraction. Results will be presented in tabular and graphical form and reported descriptively.

Ethics and dissemination: This research is exempt from ethics approval due to the data being available through published and public available resources. Results will be published in a peer-reviewed sports and exercise medicine journal regardless of positive or negative findings. In addition, results will be disseminated through multiple platforms including

For peer review only - http://bmjopen.bmj.com/site/about/guidelines.xhtml

conference presentations and social media using multimedia resources (e.g. infographics, animations, videos, podcasts, and blogs) to engage stakeholder groups including cricketers, cricket coaches, sporting bodies, sports medicine professionals and policy makers. There findings will inform clinical decision making, policy changes, and future research agendas.

Key Words: Physical Activity, Health Related Quality of Life, Injury, Mental Health, Musculoskeletal

Strengths and Limitations

- Scoping reviews are a scientifically validated method to answer broad research questions, and is the best methodology to provide an overview of all literature investigating the relationship between cricket participation, health and wellbeing.
- This scoping review will include grey literature to increase the scope and breadth of the review and screening process.
- Individual article data will be meta-aggregated to explore emergent themes, with potential to provide new insights and inform future research
- Specific articles cannot be analyzed for methodological risk of bias, decreasing the interpretability of the results.

For peer review only

For peer review only - http://bmjopen.bmj.com/site/about/guidelines.xhtml

Introduction

Cricket is a popular sport with approximately 2.5 billion people of all ages and abilities participating.[1] Cricket is played by 1.4 million people in Australia,[2] nearly 300,000 people in the United Kingdom,[3] and over 5 million people in India.[4] Further, cricket is popular among youth,[5 6] many of whom continue to play cricket into adulthood.[7] Cricket has also become increasingly popular among females with more than 27% of all Australian cricketers being female.[2] Cricket is played with 11 individuals per team, over 5 days (test cricket), one day (50 overs) or over four hours (Twenty20). The 2011 Compendium of Physical Activities list cricket as a sport that can provide moderate-intensity physical activity.[8] Regular physical activity is an important determinant of general health, life expectancy[9] and overall wellbeing.[10]

Over 31% of all adults worldwide are physically inactive, with physical inactivity levels ranging from 17% in Asia, to 43% in North America.[11] To counteract inactivity, sports participation is promoted.[12] Sport participation provides opportunities to be physically active across the lifespan.[13 14] Cricket participation can improve fitness[15 16] and strength,[16] and has psychological benefits for participants[17-19] including improved self-esteem, social connections and overall wellbeing.[17] Mental health and health-related quality of life (HRQoL) is higher in cricketers compared to the general population.[20 21] However, cricket participation is associated with injury[22-25] which can result in persistent joint pain and post-traumatic osteoarthritis.[26] Specifically, injury incidences has been reported to be up to 53 injuries per 10,000 athlete exposures,[27] with former cricketer reporting greater osteoarthritis compared to former rugby players.[28] Further, some cricketers experience increased levels of stress[29 30] and depression,[31 32] which can negatively impact HRQoL,[26 33 34] Thus, cricket participation may have both positive and negative impacts on health and wellbeing. Due to

For peer review only - http://bmjopen.bmj.com/site/about/guidelines.xhtml

the high rate of global physical inactivity,[11] the worldwide popularity of cricket,[2-4] and its viability as an outlet for physical activity across the lifespan,[13 14] information regarding the potential risks and benefits of participation in specific sports is needed to enable informed decision making for participants.

The link between sport participation and beneficial health outcomes have been synthesised in previous systematic reviews for golf, cycling, and sport and dance.[35-37] These studies found that participation in these activities had a positive relationship with physical health and wellbeing.[35-37] However, the relationship between cricket, health and wellbeing has not been investigated. Thus, there is a need to map the current evidence related to cricket, health and wellbeing, and identify key research priorities. This overview would also enable key stakeholders (including cricket participants, health professionals and sporting bodies) to make evidence informed decisions relating to cricket participation. Specifically, these data will inform stakeholders on the health and wellbeing risks and benefits of cricket participation in order to make individual and organizational decisions on the viability of promoting cricket participation as a health enhancing form of physical activity at different standards-of-play and for different age-groups. Further, identifying the gaps in the literature will allow specific cricket related research to be initiated to improve cricket participant health and wellbeing. Therefore, the aim of this scoping review is to (1) investigate the relationship between cricket participation, health and wellbeing at all ages and standards-of-play; (2) identify research gaps in the existing literature on cricket, health and wellbeing.

Methods

Patient and Public Involvement

For peer review only - http://bmjopen.bmj.com/site/about/guidelines.xhtml

No patients were involved in the design or planning of this study. However, findings from two qualitative studies investigating the relationship of physical activity and quality of life in former elite cricketers,[38 39] highlighted a need for further research investigating the relationship between cricket participation and health. It was determined that a scoping review would provide valuable information regarding the relationship between cricket participation, health and wellbeing, whilst identifying key knowledge gaps to guide future research agendas. An international stakeholder group will be established, comprising current and former cricketers, cricket coaches, sports medicine professionals, cricket-related researchers and representatives from cricket sporting bodies. This key stakeholder group will meet virtually, to discuss preliminary results and interpretation of findings, review a draft of the manuscript, and provide input into the plan for dissemination of research findings.

Study Design

The purpose of a scoping review is to describe all available evidence underpinning a given research question drawing upon research from all possible sources, consequently scoping reviews are broad in nature. This is in comparison to a systematic review which can only investigate one specific topic; as a result, a scoping review methodology was commenced.[40 41] The framework adopted for this scoping review follows existing best practice methodology.[40-44] The methodology was guided by the recommended five-stage process: identify the research question; identify relevant studies; select articles using *a priori* inclusion/exclusion criteria; chart data; collate, summarize, and report results.[40-42] The proposed study is planned to be commenced during September 2019, and is estimated to conclude in March 2020.

<u>**Stage 1: Identify the research question**</u>

For peer review only - http://bmjopen.bmj.com/site/about/guidelines.xhtml

The general research question was developed through exploration of the literature, multi-disciplinary group discussions and collaborating with experts in cricket. To reflect the context, content and the population included in the review,[40 41] the following broad research question was proposed: *what is known about the relationship between cricket participation, health and wellbeing?*

Stage 2: Identify relevant studies

A preliminary search to identify key words and index terms

A preliminary search was conducted on the major clinical and grey literature databases.[40-42] Databases included Medline, Google Scholar, and ProQuest Dissertations and Theses Global. Consistent with previous studies[42] exploratory search terms were kept broad, to be as inclusive as possible. Search terms included "*cricket*," "*health*," and "*review*." The exploratory search found 37 articles in Medline. The first 200 articles in Google Scholar were searched. Twenty-eight articles were identified as pertinent from Medline and Google Scholar. No relevant articles were found in ProQuest. These 28 articles references were then searched for further relevant articles.

The titles and abstracts of these 28 articles were then analysed for relevant search terms. The preliminary search identified a large number of irrelevant studies involving cricket insects, cadaveric, or in vitro investigations, consequently the search terms were updated to exclude articles with cadaver* or "in situ" or "in vitro" or "insects" in the title and/or abstract. The final search strategy was created to keep the search broad for greatest inclusion, while excluding specific irrelevant studies identified through the preliminary search. A medical librarian assisted by ensuring the search syntax was appropriate for each database.

For peer review only - http://bmjopen.bmj.com/site/about/guidelines.xhtml

Search strategy

Ten databases (Medline, CINAHL, Embase, Scopus, PsycINFO, SPORTDiscus, Cochrane Library, EBSCO, Web of Science and PEDro) will be electronically searched. Google Scholar, Clinicaltrials.gov, ISRCTN Registry and Proquest Dissertation and Theses will be searched for grey literature. The search strategy will be as follows, *"cricket** NOT (*cadaver* or "in situ" or "in vitro" or animals or insects*)*"* (Appendix 1) . Articles will be tracked in EndNote® X9 (Clarivate Analytics, 2018).

Study eligibility criteria

Please refer to Table 1 for inclusion and exclusion criteria.

Table 1. Inclusion and exclusion criteria

Inclusion Criteria	Exclusion Criteria
• Assesses a construct related to health (e.g. injury, pain, physiological function, physical activity, tobacco use, alcohol use, body mass index, nutrition, diabetes, cardiovascular disease) and/or wellbeing (e.g. mental health, depression, mood, anxiety, health related quality of life, resilience) in current and/or former cricketers (of any age, sex or competition level)	• Cricket performance parameters (e.g. bowling speed, wins and losses, and bowling average)
• Primary research studies, reviews, meta-analyses, guidelines or grey literature (including unpublished and ongoing trials, annual reports, dissertations and conference abstracts)	• Biomechanics (force, torque, kinematics and electromyography), joint range of motion/flexibility
• Human studies	• Cadaveric, or in situ models studies
• Articles published in English	• Editorials, periodicals, letters to the editor

For peer review only - http://bmjopen.bmj.com/site/about/guidelines.xhtml

Stage 3: Study selection

Titles and abstracts will be screened by the lead author (GSB) for eligibility, and full-text articles will be retrieved and screened by the same author (GSB) against the inclusion/exclusion criteria. A second author (NKPP) will complete the same screening process on a random sample of 10% of the articles.[42] Any title and abstract screening disputes will be resolved through the consensus of the two authors. If concordance is less than 90%, the full title and abstract screening will be performed by the second author (NKPP). Following title and abstract screening, the full text of all potentially eligible articles will be retrieved. Firstly, we will attempt to access articles through university online library portals. The online library portals will be available through collaborating institutions in the United Kingdom, Sweden, Australia, and the United States. If the article cannot be retrieved through the university online library portals, the authors will be contacted to request full-text, and if required inter library loan with the assistance of a librarian will be attempted. If a full-text article cannot be retrieved following consultation with a librarian, it will be excluded from the review.[42] If there are any discrepancies following full-text screening, a third author (SRF) will arbitrate all disputes, and decide on final article inclusion.

Stage 4: Data extraction

Data extraction procedures with follow best systematic review practice guidelines.[45] Data will be extracted by the lead author (GSB), and inputted into a customized electronic database. The customized electronic database will be based on the National Institute for Health and Care Excellence (NICE) evidence tables.[46] Quantitative data that will be extracted will include publication year, study type (primary, secondary, or grey literature), country of origin, age group, competition level, study design, study description, surgical procedure (if applicable), analysis design, and key findings. Qualitative data will be extracted through qualitative synthesis of related topics.[45] A second author (NKPP) will perform data extraction

For peer review only - http://bmjopen.bmj.com/site/about/guidelines.xhtml

on 10% of studies, selected at random. Any discrepancies in data extraction between reviewers will result in the second reviewer (NKPP) extracting data from all studies. Following this, extracted data will be cross-checked for discrepancies, and any differences in data will be resolved between reviewers. Outcome data will be stratified into *a priori* themes of musculoskeletal health, general health, and wellbeing.

Stage 5: Collating, summarizing and reporting the results

Descriptive data and key findings will be collated and summarized for descriptive analysis and the results will be presented numerically and thematically. Individual specific study quantitative and qualitative data and thematic data will be grouped into each *a priori* theme. Following the grouping of articles into each *a priori* theme, individual article data will be meta-aggregated to explore potential emergent themes.[45] Specifically, quantitative data will be extracted, sorted into relevant themes (e.g. musculoskeletal health, mental health, physiological health), and descriptively reported.[45] Qualitative data will be synthesized in excel, through a six stage process. This six stage process includes becoming familiar with the data, generating initial codes, searching for themes, reviewing themes, defining themes, and writing up. [47 48]Research gaps will be explored and tabulated through *a priori* theme and emergent theme collation. Specific article data will be tabulated and pertinent information will be aggregated into overall study data range for summarisation. Scoping review results will be presented in numeric and graphical representation for year of publication, geographic origin of publication, and *a priori* themes. A flow chart will be created to visually detail the screening and review process.[43] Emergent themes will be presented in tabular format, along with a narrative description of results.

Ethics and Dissemination

For peer review only - http://bmjopen.bmj.com/site/about/guidelines.xhtml

This research is exempt from ethical approval since it is a review of previously published articles.

This scoping review of cricket health and wellbeing is novel and will provide an overview of associations between cricket, health and wellbeing. Further, key research priorities relevant to stakeholders in cricket, including policymakers and sports governing bodies, will be clarified by this work.

Results will be published in a peer-reviewed sports and exercise medicine journal, with open access to increase information dissemination, regardless of positive or negative findings of the relationship between cricket participation, health, and wellbeing. In order to enhance knowledge translation of the findings, a multi-modal approach will be used for dissemination. Findings will be presented at conferences, multimedia resources will be (e.g. infographics, animations, videos, podcasts and blogs) will be created to disseminate findings via various social media platforms and through media release.

Conclusion

The aims and methodological study design were created in concordance with cricket stakeholders (current and former cricketers, physicians, physiotherapists, and governing bodies) in order to have a greater understanding of the relationship between cricket participation, health and wellbeing. Scoping reviews are a scientifically validated method to answer broad research questions, and summarise the knowledge gaps in this field. This scoping review will inform individuals and other stakeholders about the risk and benefits of cricket participation at all ages and standards-of-play. These findings may inform clinical decision making, policy changes, and future research agendas.

For peer review only - http://bmjopen.bmj.com/site/about/guidelines.xhtml

Author's Contributions

GSB, NPP, NA, and SRF conceived the study idea. GSB, NPP, AM, NA, and SRF were involved in methodological design and planning. GSB, NPP, and SRF wrote the first draft of the manuscript. GSB, NPP, AM, NA, and SRF all critically revised the manuscript. GSB, NPP, AM, NA, and SRF all approved the final version of the manuscript.

Funding Statement

This study was funded by Centre for Sport, Exercise and Osteoarthritis Research *Versus Arthritis* (grant reference 21595). The Centre for Sport, Exercise and Osteoarthritis Research *Versus Arthritis* had no role in study design, data collection, data analysis and interpretation, manuscript preparation, nor in the decision to submit the paper for publication.

For peer review only

References

1. International Cricket Council. ICC Members 2019 [cited 2019 4 March]. Available from: https://www.icc-cricket.com/about/members/about-our-members.
2. Network C. Record numbers playing cricket: CA. 2017. https://www.cricket.com.au/news/national-cricket-census-play-cricket-facilities-audit-james-sutherland-2016-17/2017-08-11.
3. Statista. Number of people participating in cricket in England from 2016 to 2018 2019 [Available from: https://www.statista.com/statistics/899199/cricket-participation-uk/ accessed May 6 2019.
4. Statista. India: What Sports Do You Regularly Participate In? 2019 [Available from: https://www.statista.com/statistics/562694/india-regular-participation-in-sports-by-type/ accessed May 7 2019.
5. Statistics ABO. Children's participation in cultural and leisure activities. *Canberra, Australian Bureau of Statistics* 2000
6. Maher C, Olds T, Dollman J. Adolescent sport in Australia: who, when, where and what? *ACHPER Australia Healthy Lifestyles Journal* 2009;56(1):11.
7. Bélanger M, Townsend N, Foster C. Age-related differences in physical activity profiles of English adults. *Preventive medicine* 2011;52(3-4):247-49.
8. Ainsworth BE, Haskell WL, Herrmann SD, et al. 2011 Compendium of Physical Activities: a second update of codes and MET values. *Medicine & science in sports & exercise* 2011;43(8):1575-81.
9. Warburton DE, Nicol CW, Bredin SS. Health benefits of physical activity: the evidence. *CMAJ : Canadian Medical Association journal = journal de l'Association medicale canadienne* 2006;174(6):801-9. doi: 10.1503/cmaj.051351 [published Online First: 2006/03/15]
10. Penedo FJ, Dahn JR. Exercise and well-being: a review of mental and physical health benefits associated with physical activity. *Current opinion in psychiatry* 2005;18(2):189-93.
11. Hallal PC, Andersen LB, Bull FC, et al. Global physical activity levels: surveillance progress, pitfalls, and prospects. *The lancet* 2012;380(9838):247-57.
12. Organization WH. Global action plan on physical activity 2018–2030: more active people for a healthier world: World Health Organization 2018.
13. Scheerder J, Thomis M, Vanreusel B, et al. Sports participation among females from adolescence to adulthood: A longitudinal study. *International review for the sociology of sport* 2006;41(3-4):413-30.
14. Schiphorst C, Murray A, Kelly P, et al. Infographic. Best investments for physical activity. *British journal of sports medicine* 2017;51(16):1227.
15. Noakes T, Durandt J. Physiological requirements of cricket. *Journal of Sports Sciences* 2000;18(12):919-29.
16. Johnstone JA, Ford PA. Physiologic profile of professional cricketers. *Journal of strength and conditioning research* 2010;24(11):2900-7. doi: 10.1519/JSC.0b013e3181bac3a7 [published Online First: 2010/10/27]
17. Eime RM, Young JA, Harvey JT, et al. A systematic review of the psychological and social benefits of participation in sport for children and adolescents: informing development of a conceptual model of health through sport. *The international*

For peer review only - http://bmjopen.bmj.com/site/about/guidelines.xhtml

journal of behavioral nutrition and physical activity 2013;10:98. doi: 10.1186/1479-5868-10-98 [published Online First: 2013/08/16]

18. Marlier M, Van Dyck D, Cardon G, et al. Interrelation of Sport Participation, Physical Activity, Social Capital and Mental Health in Disadvantaged Communities: A SEM-Analysis. *PloS one* 2015;10(10):e0140196. doi: 10.1371/journal.pone.0140196 [published Online First: 2015/10/10]
19. Jewett R, Sabiston CM, Brunet J, et al. School sport participation during adolescence and mental health in early adulthood. *The Journal of adolescent health : official publication of the Society for Adolescent Medicine* 2014;55(5):640-4. doi: 10.1016/j.jadohealth.2014.04.018 [published Online First: 2014/06/15]
20. Swann C, Telenta J, Draper G, et al. Youth sport as a context for supporting mental health: Adolescent male perspectives. *Psychology of sport and exercise* 2018;35:55-64.
21. Vella SA, Cliff DP, Magee CA, et al. Sports participation and parent-reported health-related quality of life in children: longitudinal associations. *The Journal of pediatrics* 2014;164(6):1469-74.
22. Soomro N, Strasiotto L, Sawdagar T, et al. Cricket Injury Epidemiology in the Twenty-First Century: What is the Burden? *Sports medicine (Auckland, NZ)* 2018;48(10):2301-16. doi: 10.1007/s40279-018-0960-y [published Online First: 2018/07/19]
23. Soomro N, Redrup D, Evens C, et al. Injury rate and patterns of Sydney grade cricketers: a prospective study of injuries in 408 cricketers. *Postgraduate medical journal* 2018;94(1114):425-31. doi: 10.1136/postgradmedj-2018-135861 [published Online First: 2018/07/28]
24. Dennis R, Farhart P, Goumas C, et al. Bowling workload and the risk of injury in elite cricket fast bowlers. *Journal of science and medicine in sport* 2003;6(3):359-67. [published Online First: 2003/11/12]
25. Orchard JW, Blanch P, Paoloni J, et al. Cricket fast bowling workload patterns as risk factors for tendon, muscle, bone and joint injuries. *British journal of sports medicine* 2015;49(16):1064-8. doi: 10.1136/bjsports-2014-093683 [published Online First: 2015/03/11]
26. Jones ME, Davies MA, Leyland KM, et al. The association of previous injury with joint pain, osteoarthritis, and joint replacement across all joints in former elite English cricketers. *Osteoarthritis and Cartilage* 2017;25 (Supplement 1):S200-S01.
27. Pardiwala DN, Rao NN, Varshney AV. Injuries in Cricket. *Sports health* 2018;10(3):217-22. doi: 10.1177/1941738117732318 [published Online First: 2017/10/04]
28. Jones ME, Davies MAM, Shah K, et al. The prevalence of hand and wrist osteoarthritis in elite former cricket and rugby union players. *Journal of science and medicine in sport* 2019 doi: 10.1016/j.jsams.2019.03.004 [published Online First: 2019/04/04]
29. Neil R, Bowles HCR, Fleming S, et al. The Experience of Competition Stress and Emotions in Cricket. *Sport Psychologist* 2016;30(1):76-88. doi: 10.1123/tsp.2014-0077
30. Hundertmark J. Cricketers and mental health concerns. *Australasian psychiatry : bulletin of Royal Australian and New Zealand College of Psychiatrists* 2007;15(6):509-12. doi: 10.1080/10398560701458210 [published Online First: 2007/09/14]
31. Jones ME, Davies MAM, Leyland KM, et al. Osteoarthritis and other long-term health conditions in former elite cricketers. *Journal of science and medicine in sport* 2018;21(6):558-63. doi: 10.1016/j.jsams.2017.10.013 [published Online First: 2017/11/02]

For peer review only - http://bmjopen.bmj.com/site/about/guidelines.xhtml

32. Sahni M, Bhogal G. Anxiety, depression and perceived sporting performance among professional cricket players. *British journal of sports medicine* 2017 doi: 10.1136/bjsports-2017-097827.5 [published Online First: 2017/05/12]
33. Schuring N, Kerkhoffs G, Gray J, et al. The mental wellbeing of current and retired professional cricketers: an observational prospective cohort study. *The Physician and sportsmedicine* 2017;45(4):463-69. doi: 10.1080/00913847.2017.1386069 [published Online First: 2017/09/28]
34. Schuring N, Aoki H, Gray J, et al. Osteoarthritis is associated with symptoms of common mental disorders among former elite athletes. *Knee surgery, sports traumatology, arthroscopy : official journal of the ESSKA* 2017;25(10):3179-85. doi: 10.1007/s00167-016-4255-2 [published Online First: 2016/08/05]
35. Oja P, Titze S, Kokko S, et al. Health benefits of different sport disciplines for adults: systematic review of observational and intervention studies with meta-analysis. *Br J Sports Med* 2015;49(7):434-40.
36. Oja P, Titze S, Bauman A, et al. Health benefits of cycling: a systematic review. *Scandinavian journal of medicine & science in sports* 2011;21(4):496-509.
37. Mansfield L, Kay T, Meads C, et al. Sport and dance interventions for healthy young people (15–24 years) to promote subjective well-being: a systematic review. *BMJ open* 2018;8(7):e020959.
38. Filbay SR, Bishop F, Peirce N, et al. Common attributes in retired professional cricketers that may enhance or hinder quality of life after retirement: a qualitative study. *BMJ open* 2017;7(7):e016541. doi: 10.1136/bmjopen-2017-016541 [published Online First: 2017/07/29]
39. Filbay SR, Bishop FL, Peirce N, et al. Physical activity in former elite cricketers and strategies for promoting physical activity after retirement from cricket: a qualitative study. *BMJ open* 2017;7(11):e017785. doi: 10.1136/bmjopen-2017-017785 [published Online First: 2017/11/21]
40. Arksey H, O'Malley L. Scoping studies: towards a methodological framework. *International journal of social research methodology* 2005;8(1):19-32.
41. Levac D, Colquhoun H, O'Brien KK. Scoping studies: advancing the methodology. *Implementation science* 2010;5(1):69.
42. Murray AD, Daines L, Archibald D, et al. The relationships between golf and health: a scoping review. *British journal of sports medicine* 2017;51(1):12-19. doi: 10.1136/bjsports-2016-096625 [published Online First: 2016/10/05]
43. Peters MD, Godfrey CM, Khalil H, et al. Guidance for conducting systematic scoping reviews. *International journal of evidence-based healthcare* 2015;13(3):141-46.
44. Murray A, Daines L, Archibald D, et al. The relationship and effects of golf on physical and mental health: a scoping review protocol. *Br J Sports Med* 2016;50(11):647-50.
45. Munn Z, Tufanaru C, Aromataris E. JBI's systematic reviews: data extraction and synthesis. *AJN The American Journal of Nursing* 2014;114(7):49-54.
46. Conaghan PG, Dickson J, Grant RL. Care and management of osteoarthritis in adults: summary of NICE guidance. *Bmj* 2008;336(7642):502-03.
47. Bree RT, Gallagher G. Using Microsoft Excel to code and thematically analyse qualitative data: a simple, cost-effective approach. *AISHE-J: The All Ireland Journal of Teaching and Learning in Higher Education* 2016;8(2)

For peer review only - http://bmjopen.bmj.com/site/about/guidelines.xhtml

48. Maguire M, Delahunt B. Doing a thematic analysis: A practical, step-by-step guide for learning and teaching scholars. *AISHE-J: The All Ireland Journal of Teaching and Learning in Higher Education* 2017;9(3)

For peer review only

Appendix ##: Example of full search strategy.

Medline and PubMed:
*cricket** NOT (*cadaver* or "in situ" or "in vitro" or animals or insects*)

For peer review only

Preferred Reporting Items for Systematic reviews and Meta-Analyses extension for Scoping Reviews (PRISMA-ScR) Checklist

SECTION	ITEM	PRISMA-ScR CHECKLIST ITEM	REPORTED ON PAGE #
**TITLE**			
Title	1	Identify the report as a scoping review.	1
**ABSTRACT**			
Structured summary	2	Provide a structured summary that includes (as applicable): background, objectives, eligibility criteria, sources of evidence, charting methods, results, and conclusions that relate to the review questions and objectives.	2
**INTRODUCTION**			
Rationale	3	Describe the rationale for the review in the context of what is already known. Explain why the review questions/objectives lend themselves to a scoping review approach.	4-5
Objectives	4	Provide an explicit statement of the questions and objectives being addressed with reference to their key elements (e.g., population or participants, concepts, and context) or other relevant key elements used to conceptualize the review questions and/or objectives.	5
**METHODS**			
Protocol and registration	5	Indicate whether a review protocol exists; state if and where it can be accessed (e.g., a Web address); and if available, provide registration information, including the registration number.	This is the prot ocol
Eligibility criteria	6	Specify characteristics of the sources of evidence used as eligibility criteria (e.g., years considered, language, and publication status), and provide a rationale.	7-8
Information sources*	7	Describe all information sources in the search (e.g., databases with dates of coverage and contact with authors to identify additional sources), as well as the date the most recent search was executed.	6-7
Search	8	Present the full electronic search strategy for at least 1 database, including any limits used, such that it could be repeated.	6-7
Selection of sources of evidence†	9	State the process for selecting sources of evidence (i.e., screening and eligibility) included in the scoping review.	8
Data charting process‡	10	Describe the methods of charting data from the included sources of evidence (e.g., calibrated forms or forms that have been tested by the team before their use, and whether data charting was done independently or in duplicate) and any processes for obtaining and confirming data from investigators.	8-9
Data items	11	List and define all variables for which data were sought and any assumptions and simplifications made.	9
Critical appraisal of individual sources of evidence§	12	If done, provide a rationale for conducting a critical appraisal of included sources of evidence; describe the methods used and how this information was used in any data synthesis (if appropriate).	Only study met hodology will b e appraised
Synthesis of results	13	Describe the methods of handling and summarizing the data that were charted.	9

St. Michael's
Inspired Care. Inspiring Science.

For peer review only - http://bmjopen.bmj.com/site/about/guidelines.xhtml

SECTION	ITEM	PRISMA-ScR CHECKLIST ITEM	REPORTED ON PAGE #
**RESULTS**			
Selection of sources of evidence	14	Give numbers of sources of evidence screened, assessed for eligibility, and included in the review, with reasons for exclusions at each stage, ideally using a flow diagram.	Protocol
Characteristics of sources of evidence	15	For each source of evidence, present characteristics for which data were charted and provide the citations.	Protocol
Critical appraisal within sources of evidence	16	If done, present data on critical appraisal of included sources of evidence (see item 12).	Protocol
Results of individual sources of evidence	17	For each included source of evidence, present the relevant data that were charted that relate to the review questions and objectives.	Protocol
Synthesis of results	18	Summarize and/or present the charting results as they relate to the review questions and objectives.	Protocol
**DISCUSSION**			
Summary of evidence	19	Summarize the main results (including an overview of concepts, themes, and types of evidence available), link to the review questions and objectives, and consider the relevance to key groups.	Protocol
Limitations	20	Discuss the limitations of the scoping review process.	Protocol
Conclusions	21	Provide a general interpretation of the results with respect to the review questions and objectives, as well as potential implications and/or next steps.	Protocol
**FUNDING**			
Funding	22	Describe sources of funding for the included sources of evidence, as well as sources of funding for the scoping review. Describe the role of the funders of the scoping review.	12

JBI = Joanna Briggs Institute; PRISMA-ScR = Preferred Reporting Items for Systematic reviews and Meta-Analyses extension for Scoping Reviews.
* Where *sources of evidence* (see second footnote) are compiled from, such as bibliographic databases, social media platforms, and Web sites.
† A more inclusive/heterogeneous term used to account for the different types of evidence or data sources (e.g., quantitative and/or qualitative research, expert opinion, and policy documents) that may be eligible in a scoping review as opposed to only studies. This is not to be confused with *information sources* (see first footnote).
‡ The frameworks by Arksey and O'Malley (6) and Levac and colleagues (7) and the JBI guidance (4, 5) refer to the process of data extraction in a scoping review as data charting.
§ The process of systematically examining research evidence to assess its validity, results, and relevance before using it to inform a decision. This term is used for items 12 and 19 instead of "risk of bias" (which is more applicable to systematic reviews of interventions) to include and acknowledge the various sources of evidence that may be used in a scoping review (e.g., quantitative and/or qualitative research, expert opinion, and policy document).

From: Tricco AC, Lillie E, Zarin W, O'Brien KK, Colquhoun H, Levac D, et al. PRISMA Extension for Scoping Reviews (PRISMA-ScR): Checklist and Explanation. Ann Intern Med. ;169:467–473. doi: 10.7326/M18-0850

For peer review only - http://bmjopen.bmj.com/site/about/guidelines.xhtml
